# Transcriptome and proteome analyses of adaptive responses to methyl methanesulfonate in *Escherichia coli *K-12 and *ada *mutant strains

**DOI:** 10.1186/1471-2180-9-186

**Published:** 2009-09-03

**Authors:** Jong Hwan Baek, Mee-Jung Han, Sang Yup Lee, Jong-Shin Yoo

**Affiliations:** 1 Metabolic and Biomolecular Engineering National Research Laboratory, Department of Chemical & Biomolecular Engineering (BK21 Program), BioProcess Engineering Research Center, Center for Systems and Synthetic Biotechnology and Institute for the BioCentury, KAIST, 335 Gwahangno, Yuseong-gu, Daejeon 305-701, Republic of Korea; 2 Laboratory of Biochemistry and Molecular Biology, Center for Cancer Research, National Cancer Institute, National Institutes of Health, Bethesda, MD 20892, USA; 3 Department of Chemical & Biomolecular Engineering, Dongyang University, # 1 Gyochon-dong, Punggi-eup, Yeongju, Gyeongbuk 750-711, Republic of Korea; 4 Department of Bio and Brain Engineering, Department of Biological Sciences and Bioinformatics Research Center, KAIST, Republic of Korea; 5 Mass Spectrometry Research Center, Korea Basic Science Institute, 804-1 Yangcheong-Ri Ochang-Eup, Cheongwon-Gun, Chungbuk 863-883, Republic of Korea

## Abstract

**Background:**

The Ada-dependent adaptive response system in *Escherichia coli *is important for increasing resistance to alkylation damage. However, the global transcriptional and translational changes during this response have not been reported. Here we present time-dependent global gene and protein expression profiles following treatment with methyl methanesulfonate (MMS) in *E. coli *W3110 and its *ada *mutant strains.

**Results:**

Transcriptome profiling showed that 1138 and 2177 genes were differentially expressed in response to MMS treatment in the wild-type and mutant strains, respectively. A total of 81 protein spots representing 76 nonredundant proteins differentially expressed were identified using 2-DE and LC-MS/MS. In the wild-type strain, many genes were differentially expressed upon long-exposure to MMS, due to both adaptive responses and stationary phase responses. In the *ada *mutant strain, the genes involved in DNA replication, recombination, modification and repair were up-regulated 0.5 h after MMS treatment, indicating its connection to the SOS and other DNA repair systems. Interestingly, expression of the genes involved in flagellar biosynthesis, chemotaxis, and two-component regulatory systems related to drug or antibiotic resistance, was found to be controlled by Ada.

**Conclusion:**

These results show in detail the regulatory components and pathways controlling adaptive response and how the related genes including the Ada regulon are expressed with this response.

## Background

Alkylation damage to DNA occurs when cells encounter alkylating agents in the environment or when active alkylators are generated by nitrosation of amino acids in metabolic pathways [1,2]. The DNA damage by alkylating agents results in disruption of DNA function and cell death. The alkylating agents represent an abundant class of chemical DNA damaging agent in our environment and are toxic, mutagenic, teratogenic and carcinogenic. Since we are continuously exposed to alkylating agents, and since certain alkylating agents are used for cancer chemotherapy, it is important to understand exactly how cells respond to these agents. Alkylating agents are commonly used anti-cancer drugs and remain important for the treatment of several types of cancer [3,4]. Alkylating drugs are mostly methylating agents (e.g. temozolomide and streptozotocin, an antibiotic) or chloroethylating agents (e.g. carmustine, lomustine and fotemustine) [5]. The efficacies of these drugs are strongly modulated by DNA repair process. Thus, thorough understanding and controlling of the repair processes will allow development of new therapies by protecting normal tissues or by potentiating effects in the target tissues.

To cope with DNA alkylation damage, cells have evolved genes that encode proteins with alkylation-specific DNA repair activities. It is notable that these repair systems are conserved from bacteria to humans [6]. In *Escherichia coli*, cells exposed to a low concentration of an alkylating agent, such as *N*-methyl-*N'*-nitro-*N*-nitrosoguanidine (MNNG) or methyl methanesulfonate (MMS), show a remarkable increase in resistance to both the lethal and mutagenic effects of subsequent high-level challenge treatments with the same or other alkylating agents [7,8]. This increased resistance has been known as "adaptive response" to alkylation damage in DNA.

To date, four genes have been identified as components of this response, *ada*, *alk*A, *alk*B and *aid*B. The *ada *gene encodes the Ada protein, which has the dual function of a transcriptional regulator for the genes involved in the adaptive response, and a methyltransferase that demethylates two methylated bases (O^6^meG and O^4^meT) and methylphosphotriesters produced by methylating agents in the sugar phosphate backbone [6,9]. When methylated at Cys-69, Ada is converted to a potent activator for the transcription of the *ada *and *alk*A, *alk*B and *aid*B genes by binding to a consensus sequence referred to as an "Ada box" present in the promoter. The *alk*A gene encodes a glycosylase that repairs several different methylated bases, and the *alk*B gene, which forms a small operon with the *ada *gene, is required for error-free replication of methylated single-stranded DNA [10]. The *aid*B gene encodes the protein that appears to detoxify nitrosoguanines and to reduce the level of methylation by alkylating agents. Early studies have shown that the expression of the *ada-alk*B operon, *alk*A and *aid*B genes is positively controlled by Ada protein, after it interacts with methylated DNA [11-14]. In contrast, Ada protein also plays a pivotal role in the negative modulation of its own synthesis, and consequently, in the down-regulation of the adaptive response. The carboxyl-terminus of Ada protein appears to be necessary for this negative regulatory function; thus, Ada protein can act as both a positive and a negative regulator for the adaptive response of *E. coli *to alkylating agents [13]. The transcriptional activity of *E. coli *Ada protein is also directly regulated by posttranslational covalent modification; however, the regulatory components and pathways controlling the adaptive response have not been well studied.

Recent advances in functional genomics studies have facilitated understanding of global metabolic and regulatory alterations caused by genotypic and/or environmental changes. DNA microarray has proven to be a successful tool for monitoring genome-wide expression profiles at the mRNA level. Similarly, proteomics can be used to compare changes in levels of many proteins under particular genetic and environmental conditions. Furthermore, proteomic studies provide information on posttranslational modifications, which cannot be obtained from mRNA expression profiles; these have proven critical to our understanding of proper physiological protein function, translocation, and subcellular localization. Ideally, information obtained from these technologies needs to be integrated to better understand the phenotypic characteristics of the cell under a given condition [15]. Recently, combined transcriptome and proteome approaches have allowed large-scale analysis of biological systems at the mRNA and protein levels, providing us with a wealth of information that is useful in data-driven discovery [16-19].

In this paper, we report the global expression changes in the gene and protein levels of *E. coli *K-12 W3110 and *ada *mutant strains in response to alkylating agents. In addition, the differences between the wild-type and mutant strains without treatment of alkylating agents were characterized at transcriptome and proteome levels. The analysis of time- and strain-dependent adaptive responses revealed the regulatory and physiological characteristics of the Ada-dependent adaptive response in *E. coli*.

## Results and discussion

### Growth profiles of *E. coli *W3110 and *ada *mutant strains under MMS-treated and -untreated conditions

**Figure 1:**
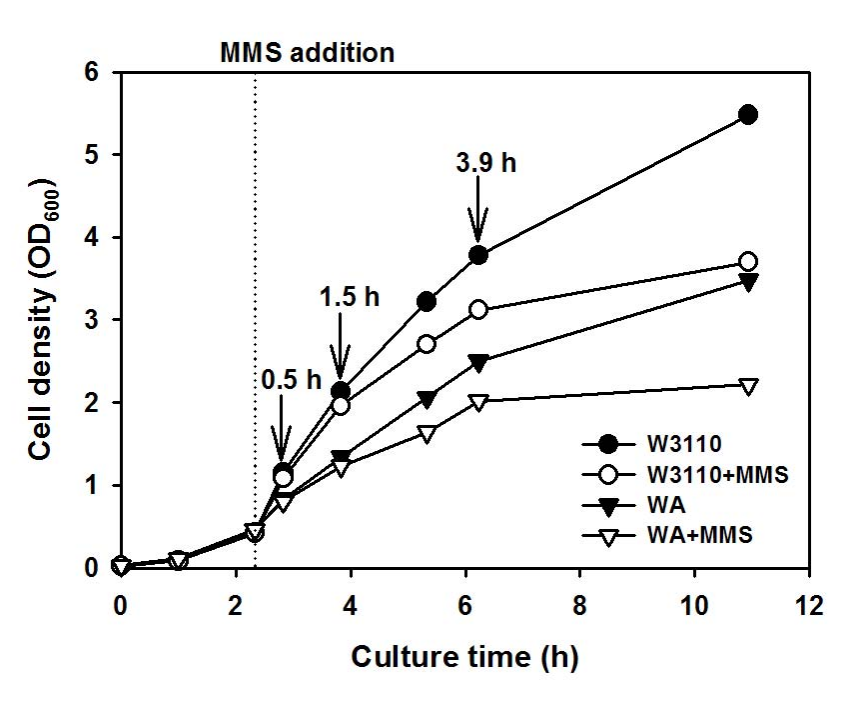
**Growth profiles of *E. coli *W3110 (circle) and its *ada *mutant (triangle) strains**. Each strain was cultivated with or without 0.04% MMS treatment (open or filled symbols, respectively) at the exponential phase (at 2.8 h; dotted line). Arrows indicate the sampling times (0.5, 1.5 and 3.9 h after MMS treatment) for transcriptome and proteome analyses.

Growth of the *ada *mutant strain was reduced in LB medium without MMS addition according to culture time, and reached the final OD_600 _of 3.48, which was about 1.5-fold lower than that of the wild-type (Figure 1). In order to induce adaptive responses that increase resistance to alkylation damage to DNA, cells were treated with 0.04% MMS at an OD_600 _of 0.4 [20]. As shown in Figure 1, the growth of both strains gradually retarded following MMS addition. The final OD_600 _of 3.70 and 2.22 were reached at 11 h for the wild-type and the *ada *mutant strains, respectively, which were significantly lower than those of the control cultures. However, there were no noticeable differences in cell size and morphology between the *ada *mutant and its parent strains. Growth of the *ada *mutant strain was found to be additionally inhibited after the MMS treatment. This indicates that the defect in the *ada *gene negatively influences cell growth even under the normal condition, and especially the *ada *product has an important role in adaptive responses when alkylating agents are present, as has been shown previously [21]. The difference of growth between the strains will be discussed later combined with transcriptome and proteome analyses. It should be noted that the last sampling points are in the middle of stationary phase for all strains with and without MMS treatment, which becomes evident when the growth curve is redrawn in log-scale.

### Transcriptome and proteome profiles of *E. coli *W3110 in response to MMS

Transcriptome and proteome analyses were performed for the samples taken at 0.5, 1.5 and 3.9 h following MMS treatment for both MMS-treated and -untreated control cultures, and the expression levels were compared. Those genes and proteins which were differentially expressed by greater than 2-fold or less than a half in MMS-treated cells compared with the controls (MMS-untreated cells) were considered to be meaningfully up- or down-regulated ones by MMS treatment.

**Figure 2:**
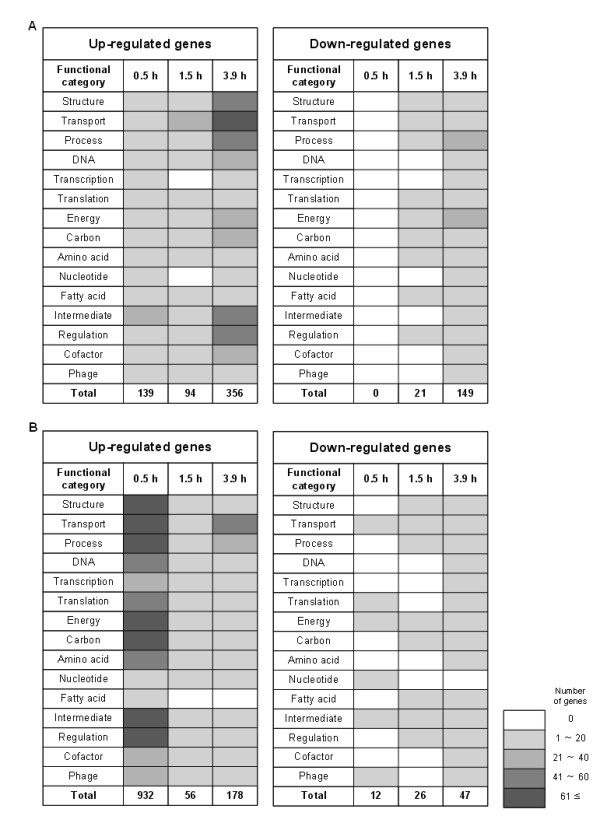
**Distribution of differentially expressed genes**. *E. coli *W3110 (A) and its *ada *mutant (B) strains at each time profile (0.5, 1.5 and 3.9 h) were sampled and compared after MMS treatment based on the corresponding untreated control. The up- or down-regulated genes at each time point were counted after classification by functional categories according to the *E. coli *genome information [22]. Categories: Structure, cell structure, membrane proteins, structural proteins; Transport, transport and binding proteins, putative transport proteins; Process, cell processes including adaptation and protection; DNA, DNA replication, recombination, modification and repair; Transcription, transcription, RNA processing and degradation; Translation, translation, post-translational modification; Energy, energy metabolism; Carbon, carbon compound catabolism; Amino acid, amino acid biosynthesis and metabolism; Nucleotide, nucleotide biosynthesis and metabolism; Fatty acid, fatty acid and phospholipid metabolism; Intermediate, central intermediary metabolism; Regulation, regulatory function, putative regulatory proteins; Cofactor, biosynthesis of cofactors, prosthetic groups and carriers; Phage, phage, transposon or plasmid. The number of total genes was indicated at the bottom of each heat map.

**Figure 3:**
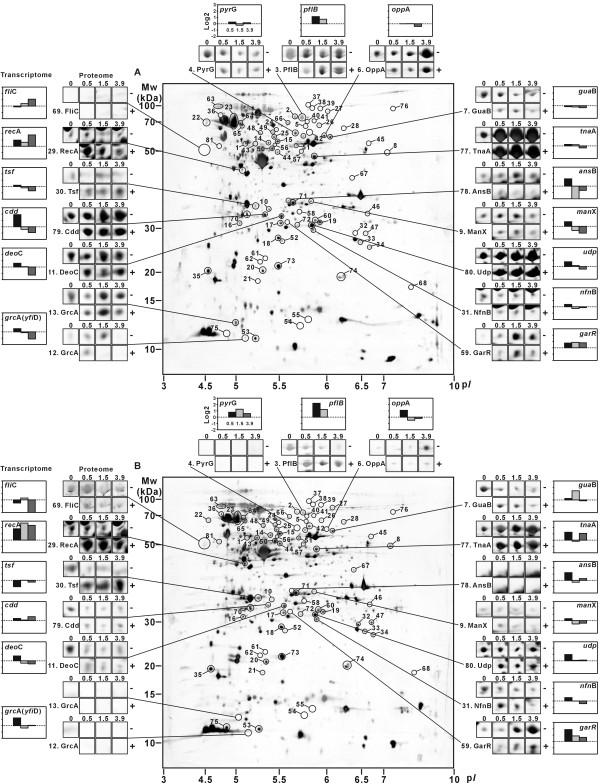
**Proteome and transcriptome profiles of *E. coli *W3110 (A) and its *ada *mutant (B) strains**. The proteins showing significantly altered levels according to exposure time of MMS are indicated on each 2-D gel as circles when samples taken from MMS-treated cells were compared to the corresponding untreated control. Of these, seventeen zoomed in areas highlighted from the 0 h profile gel of each strain are compared to corresponding protein spots of the 0.5, 1.5 and 3.9 h profile gels with (+) or without (-) MMS addition. Also, the fold difference (log2 scale) of expression level of the corresponding genes of *E. coli *W3110 (A) and *ada *mutant strains (B) under MMS-treated and -untreated conditions are shown next to the panels of proteome spots.

To find further functional characteristics of genes implicated in adaptive response, differentially expressed genes of known function were selected and classified according to functional category [22] (Figure 2). At 0.5 h following MMS treatment, 139 genes were found to be up-regulated, while no gene was down-regulated. Proteome analysis showed the induction of 17 protein spots in MMS treated cultures (Figure 3, Additional file 1: Table S1). The most strongly induced proteins were those involved in DNA replication, recombination, modification and repair (RecA and Mfd); cell process including adaptation and protection (AhpF, HtpG, NfnB and YfiD); translation and posttranslational modification (DsbA, InfB, ProS, RpsB, ThrS and one isoform of Tsf); and others (Eda, GlpD, RpoC, YjgF and YeaG). Interestingly, a different isoform of elongation factor Ts (encoded by the *tsf *gene) was detected in the case of MMS-treated cells, the spot intensity of which significantly increased with exposure time to MMS. In contrast, the total amount of this protein was not significantly changed over time similarly to the mRNA expression level (Figure 3). In addition, GrcA (Synonyms: YfiD) known to be induced by acid stress had also two isoforms (spots 12 and 13) on the 2-D gels. The response tendency of the total level of this protein was similar to that of the gene expression level (Figure 3). These results indicate that MMS treatment triggers synthesis of some proteins in different isoforms by posttranslational modification.

As expected, 13 genes involved in DNA replication, repair and modification (*ada*, *alk*B, *din*D, *mut*S, *pol*B, *rec*N, *rne*, *sbm*C, *tpr*, *tus*, *umu*D and *uvr*AB) were up-regulated to allow prevention and repair of replication-blocking lesions in *E. coli *cells exposed to alkylation stress. Among these, the genes in the Ada regulon, *ada *and *alk*B were strongly induced, which indicates that cells experiencing DNA damage in response to MMS exposure try to mend the damage by inducing the DNA repair system that is regulated by Ada. In addition to the Ada transcriptional regulator (*ada*), the expression of the genes encoding other transcriptional regulators, such as the *ara*C, *kdp*E, *mar*A, *yad*W, *yaf*C, *ybd*O and *ykg*D genes, was significantly up-regulated as seen in the 0.5 h transcriptome profiles. These regulators might influence a dynamic network of the adaptive response. The transcriptome experiments also revealed that genes related to a variety of other cell processes, including chaperones (*hsc*A and *htp*G), degradation of small molecules (*cai*BDT), and adaptation and protection (*bet*A, *gef*, *htg*A, *ibp*A and *mar*A), were induced after MMS treatment. These responses are consistent with the proteome data showing the induction of four proteins (AhpF, HtpG, NfnB and YfiD) categorized into the adaptation and protection function. Induction of these proteins seems to be involved in the protection of genes and/or proteins against MMS toxicity.

In addition, a large number of genes with altered expression levels (356 up-regulated and 149 down-regulated) was seen in 3.9 h profiles for *E. coli *W3110 cells (Figure 2). These mainly included genes involved in structure, cell process and transport. These larger changes in the expression level might be due to the complicated response in the stationary phase to alkylating agents. Previously, Sedgwick et al. [1] reported that the Ada regulon could be induced during stationary phase and could protect against active alkylators produced by nitrosation of amino acids in non-growing cells. Therefore, an increase in expression of the adaptive response genes, in parallel with expression of the genes producing active alkylators during the stationary phase prevents alkylation damage to DNA and subsequent mutagenesis.

### Transcriptome and proteome profiles of the *ada *mutant strain in response to MMS

**Figure 4:**
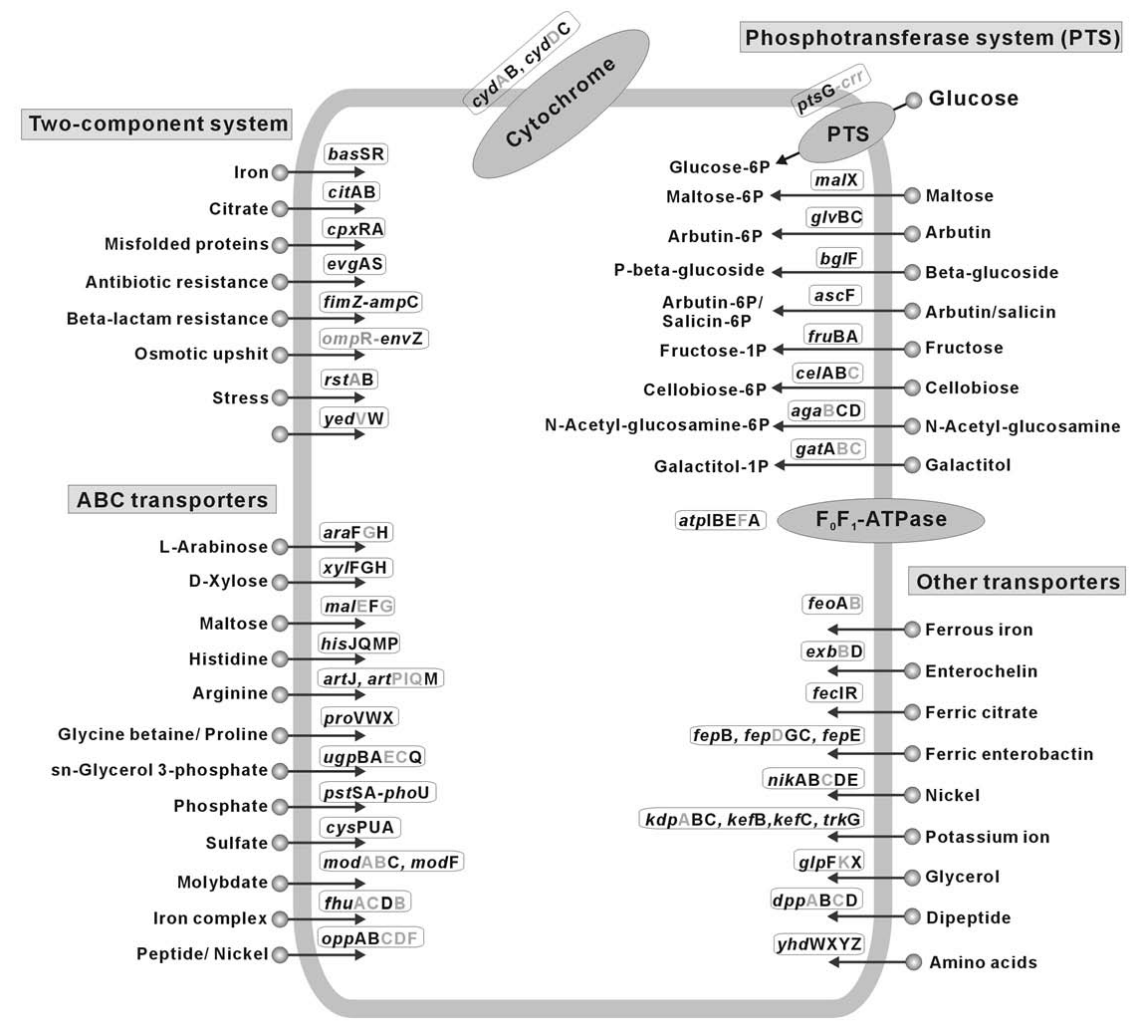
**Schematic diagram of up-regulated genes in the MMS-treated *E. coli ada *mutant strain**. The two-component system related to drug or antibiotic resistance and the operons of genes related to respiration and transport are shown. The genes up-regulated more than 2-fold by 0.5 h MMS treatment, based on the corresponding untreated control in the *ada *mutant strain, are indicated in black bold type.

The transcriptional and translational responses of the *ada *mutant strain to alkylation stress were vastly different from those observed in W3110 strain (Figure 2). In the *ada *mutant strain, the expression levels of many more genes were significantly changed at 0.5 h after MMS treatment; 932 genes were up-regulated, which was about seven-fold more than that observed in the wild-type strain. Also, 12 genes of known function were down-regulated (Figure 2). The responses of the *ada *mutant to alkylating agents revealed several common themes, including the activation of genes involved in the transport of ions, sugars and amino acids and in detoxification processes (Figure 4). This result indicates that the *ada *mutant cells induce various genes related to influx or efflux of solutes as a means of preventing and repairing alkylation damage. However, unlike the wild-type cells, in which these genes were up-regulated at 3.9 h after MMS treatment, the expression of transport genes was down-regulated in the *ada *mutant cells after the initial alkylation stress was compensated. Based on these results, it can be assumed that the transport system substitutes for the adaptive response system in the *ada *mutant strain to coordinate the instant activation of the cellular repair systems after MMS treatment. More details are described below.

Proteome analysis showed variations in the production levels of 21 protein spots; the spot intensities of 18 proteins increased while 3 proteins decreased (Figure 3, Additional file 1: Table S1). Consistent with the transcriptome data, the intensities of proteins involved in metabolism and transport were increased. Proteins that showed significantly increased spot intensities in MMS-treated *ada *mutant cells at 0.5 h included enzymes for DNA replication, recombination, modification and repair (RecA and Mfd); cell process including adaptation and protection (AhpF, CheA, HtpG and NfnB); translation and posttranslational modification (RpsB, ThrS and an isoform of Tsf); transport and binding proteins (DppA and OppA); metabolisms (FabG, GlpA, PflB, PyrG (spot no. 42) and TreC), and YeaG. Similar to proteome profiles of MMS-treated wild-type cells, one isoform of elongation factor Ts (Tsf) was detected on 2-D gels of MMS-treated *ada *cells. Interestingly, the MMS treatment of the *ada *mutant cells resulted in the significant repression of the FliC involved in flagellar biosynthesis, which is consistent with down-regulated expression of this gene in transcriptome data (Additional file 1: Table S1).

In general, *E. coli *responds to alkylation stress by activating sets of co-regulated genes that help the cell to maintain homeostasis. However, the *ada *mutant cells would require a more rapid increase in the expression levels of specific genes for DNA repair in response to methylating agents, due to the lack of the Ada-dependent response mechanism. It can be seen from the 0.5 h profiles that the adaptive response mediates the induction of 23 genes involved in DNA replication, recombination, modification and repair, such as *b1360*, *din*D, *lar, mod*F, *mut*H, *ogt*, *phr*B, *pin*O, *pol*B, *pri*A, *rec*ANT, *rnb*, *rnp*A, *ruv*B, *tpr*, *umu*CD, *uvr*A, *yee*S, *yfb*L and *yfj*Y. MMS treatment also caused a strong induction of the drug or antibiotic resistance genes, most of which are located in cell membrane (Figure 4, Additional file 2). Proteome profiles also showed that RcsB was increased in MMS-treated *ada *mutant cells. Taken together, the profiles for the *ada *mutant strain defective in adaptive response showed a far more rapid transcriptional response following MMS treatment when compared to the wild-type. From these results, we reasoned that the responses observed at earlier time point might allow identification of direct targets of the adaptive response, while the long-exposure time profiles would reflect more complex regulation in cellular networks, including both stationary phase responses by the *rpo*S gene product [23,24] and adaptive response by alkylating agents. Thus, the transcriptional and translational profiles of the wild-type and the *ada *mutant strain at 0.5 h were analyzed in more detail.

### Differences in expression levels between wild-type and *ada *mutant strains under normal growth condition

In order to examine the intracellular changes that are induced by the *ada *gene deletion in the MMS-untreated, normal growth condition, the expression levels of genes and proteins of *ada *mutant cells were first compared with those of wild-type cells at the mid-log growth phase (at 0.5 h sampling point). The number of genes differentially expressed at greater than 2-fold levels was small. Only 69 and 10 genes were up- and down-regulated, respectively, in the *ada *mutant strain compared to the wild-type strain (Additional file 2). Interestingly, the expression levels of the genes involved in flagellar biosynthesis (*flg*CEG and *fli*AC) and chemotaxis (*tar *and *che*W) were higher in the *ada *mutant strain than in the wild-type. Proteome analysis indicated 26 protein spots that were clearly affected in the *ada *mutant cells, half of them being significantly over-produced compared with the wild-type strain, while the others being under-produced (Additional file 1: Table S1). Eight proteins, including CheAY, FliC, GlnA, InfB, Mfd, OsmY and PyrG (spot no. 42), were present only in the *ada *mutant strain, while AnsB, GrcA (two spots), OppA and PyrG (spot no. 4) were detected only in the wild-type strain. Interestingly, the *ada *mutant cell showed a different isoform distribution for CTP synthase (PyrG) compared with that of the wild-type. This finding suggests that the *ada *mutation alters this protein by posttranslational modification.

Consistent with the transcriptome data, the main differences between the two strains were identified as the flagellar biosynthesis protein (FliC) and chemotaxis proteins (CheAY). These results indicate that Ada might be a negative regulator of bacterial chemotaxis under normal growth condition. In addition, the small differences between the strains suggest the limited role that Ada plays under normal growth condition. In fact, there have been no reports on any other functions of Ada except its adaptive response to protect cells from DNA damage by alkylating agents [21]. However, our study indicates that Ada plays an additional role as a transcriptional regulator under normal growth condition and this can be a reason why the final concentration of the *ada *mutant strain was lower than that of the wild-type strain, as shown in Figure 1.

### Expression levels of the genes in the Ada regulon

**Figure 5:**
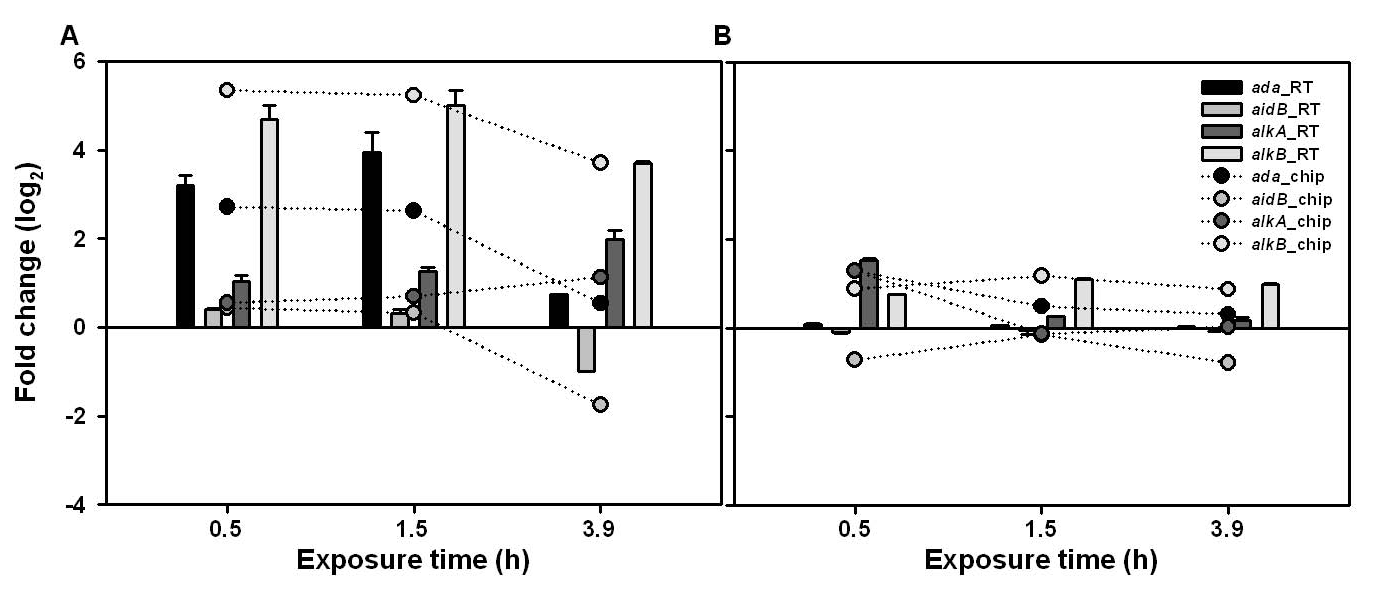
**The expression levels of the Ada regulon**. The expression levels of the *ada*, *aid*B, *alk*A and *alk*B genes of *E. coli *W3110 (A) and its *ada *mutant (B) strains at each time profile (0.5, 1.5 and 3.9 h) after MMS treatment were revealed by DNA microarray (chip) and real-time PCR (RT) analyses, compared to the corresponding untreated control. The real-time PCR experiments were conducted at least three times with independently isolated RNA sample.

As mentioned previously, the adaptive response set of genes is comprised of the *ada*, *alk*A, *alk*B and *aid*B genes [7]. Expression of these genes is regulated by Ada, and their induction provides protection against alkylation damage to DNA. To validate the expression levels of these genes in response to alkylation damage, we examined their transcriptional levels in both *E. coli *W3110 and the *ada *mutant strains at three different time points after MMS treatment, by both DNA microarray and real-time PCR analyses (Figure 5). As expected, the results obtained from real-time PCR analysis strongly correlated with those from DNA microarray analysis (r^2 ^= 0.90). The expression levels of the genes in wild-type and mutant strains were obviously different in MMS-treated condition. However, they were not significantly changed over time in the *ada *mutant strain, compared with those of its parent strain. On the other hand, the *alk*A and related genes showed gradually increased or decreased expression levels over the time in the wild-type and mutant strains, respectively. This implies that the adaptive response resulting from the up-regulation of these genes was not induced in the absence of the *ada *gene. This finding is in good agreement with previous reports showing that the expression of these four genes is positively controlled by Ada only after it interacts with methylated DNA [11-14].

The expression profiles of genes involved in the adaptive response of *E. coli *could be divided into two groups: namely, *ada*-like or *alk*A-like expression profiles. The *ada*-like expressed genes including the *ada*, *alk*B and *aid*B genes showed the highest expression levels relatively early after MMS addition (at 0.5 h and 1.5 h profiles) and decreased later. On the other hand, the *alk*A-like expressed genes, such as the *alk*A gene, presented a gradually increased expression level over the time. A previous study showed that the *ada *and *alk*A genes are regulated by a distinct mechanism in response to alkylation damage [21], and this is supported by our data. However, the differences in the expression levels of the four genes (*ada*, *alk*A, *alk*B and *aid*B) between the wild-type and *ada *mutant strains were negligible under normal condition (data not shown), which suggests that this adaptive response might reflect an inducible mechanism that generates genetic variability in times of alkylation stress.

### Increased expression levels of the genes and proteins involved in flagellar biosynthesis and chemotaxis

The synthesis and proper functioning of the flagellar and chemotaxis system require the expression of more than 50 genes, which are divided among at least 17 operons constituting the large, coordinately regulated flagellar regulon [25]. As described above, even under normal growth condition, the expression levels of the genes belonging to this group were increased in the *ada *mutant strain compared to the wild-type strain, and were further increased at 0.5 h following MMS treatment. The key master regulator, encoded by *flh*CD, was moderately increased in the *ada *mutant cells at 0.5 h after MMS treatment and five additional flagellar biosynthesis genes (*flg*AH, *flh*B and *fli*ST) were also up-regulated. Four genes involved in the chemotaxis signal transduction system were up-regulated including the genes for three chemoreceptors (*aer, tar *and *trg*) and the CheA kinase (*che*A), which activates the CheY response regulator via phosphorylation and then influences flagellum activity through interaction with the motor. These findings also agree with proteomic data that showed that enzymes of chemotaxis (CheAY) and flagellar biosynthesis (FliC) were detected only in the *ada *mutant strain (Figure 3, Additional file 1: Table S1). These chemotaxis genes are not directly regulated by FlhDC, but are controlled by the flagellum sigma factor, σ^F^, encoded by *fli*A. However, the expression of *fli*A was not significantly changed with time in the *ada *mutant cells, although the *fli*AYZ operon is regulated by FlhCD. The protein kinase, CheA, plays a central role in the initial excitation responses to stimuli as well as in the subsequent events associated with adaptation. The activity of the CheA kinase is increased by the increased levels of receptor methylation [26]. High levels of receptor methylation have been correlated with tumbly behavior, providing evidence that changes in receptor methylation mediate adaptive responses to attractant and repellent stimuli. Thus, the increased expression of these genes is closely related to negative Ada-dependent regulation in *E. coli *and Ada might negatively affect the protein components of bacterial chemotaxis. The flagellar biosynthesis genes and chemotaxis genes seem to contribute to protecting the viability of *ada *mutant cells by transferring methyl group to methyl-accepting proteins (MCP) such as Aer, Tar and Trg.

### Increased expression levels of the genes and proteins related to drugs or antibiotics resistance

The *ada *mutant cells that are hypersensitive to alkylating agents compared to wild-type cells might need to activate the expression of drug or antibiotic resistance genes to reduce their susceptibility to alkylation damage. In fact, many genes involved in these functions were found to be induced, some rapidly and some later in response to MMS treatment (Figure 4). The expression level of the *fsr *gene responsible for fosmidomycin resistance was rapidly and continuously induced in both strains after MMS treatment, and this gene also showed increased expression in the *ada *mutant strain compared to the wild-type under normal growth condition. Additionally, genes encoding the multiple antibiotic resistance protein (*mar*ABR), microcin B17 uptake protein (*sbm*A), and putative resistance protein (*yde*A) were also up-regulated in both strains at 3.9 h post MMS treatment, in the stationary phase. This observation is consistent with the fact that the Ada regulon is highly induced during the stationary phase [24] and that it protects cells from active alkylators produced by nitrosation of amino acids [1,2]. However, some of genes belonging to this function showed different expression patterns between the strains. For example, the genes encoding multidrug resistance proteins (*emr*ABDE) were rapidly induced at 0.5 h profile in the *ada *mutant strain and decreased afterwards. On the other hand, some of these genes (*emr*BEY) were increased later at 3.9 h profile only in the wild-type strain. This result suggests that the *ada *mutant strain might require a timely and rapid induction of the drug or antibiotic resistance genes to reduce its susceptibility to alkylation damage. Proteome data also showed induction of proteins related to detoxification (AhpF and NfnB) in both strains following MMS treatment. Alkylating agents that target DNA-associated processes are anticipated to be far more specific and effective as antibiotics or drugs [3-5]. This seems to be why the drug or antibiotics resistance genes were expressed upon MMS treatment. In addition, it has been demonstrated that DNA repair is enhanced in drug-resistant cell lines and tumors [3]. These results indicate that the expression of drug or antibiotic resistance genes might be affected in the DNA repair processes.

In addition, proteome analysis indicated that RcsB responded to peptidoglycan damage and contributed to intrinsic antibiotic resistance of *E. coli *[27] was synthesized at high level in the *ada *mutant strain. The finding allowed us to further examine the changes of the expression levels of drug or antibiotic resistance genes from transcriptome profiles. Hirakawa *et al*. [28] demonstrated that overexpression of fifteen genes, *bae*R, *cit*B, *cpx*R, *evg*A, *fim*Z, *kdp*E, *nar*LP, *omp*R, *rcs*B, *rst*A, *tor*R, *yed*W, *yeh*T and *dcu*R, which are response regulators of two-component signal transduction systems in *E. coli*, conferred increased single- or multidrug resistance. Interestingly, as shown in Figure 4, most of these genes, including the *bae*R, *cit*B, *cpx*R, *evg*A, *fim*Z, *omp*R, *rcs*B, *rst*A and *yed*W genes, were up-regulated in the *ada *mutant strain at 0.5 h after MMS treatment. Expression of the cognate sensor gene of two-component transduction systems (*bae*RS, *cit*AB, *cpx*AR, *evg*AS and *rst*AB, but not *yed*VW) increased coordinately when it was cotranscribed with the regulator. Increased expression levels were also observed when the sensor was even in a separate operon (*fim*Z-*amp*C and *omp*R-*env*Z). However, no induction of these two-component transduction genes was observed in MMS-treated wild-type strain. These findings show that the up-regulated genes of the bacterial two-component signal transduction systems might confer MMS resistance in the absence of the *ada *gene, through the control of the expression of drug or antibiotic transporter genes [29,30]. This type of response regulator-mediated drug resistance might be required for acquiring MMS toxicity resistance although the mechanism of the response is not yet clear. Furthermore, this is closely correlated with the finding that increased expression levels of the genes involved in transport systems are seen in the 0.5 h profile of the *ada *mutant strain (Figure 4). The influx and efflux of solutes through the cell might also play a major role in intrinsic tolerance of bacteria to drugs and toxic compounds as adaptive responses.

### Induction of DNA repair mechanisms

The prevention of the mutagenic and lethal consequences of DNA damage requires the timely expression of DNA repair and protective genes, in order to maintain the integrity of the genome and viability of the cell. As pointed out before, Ada is an important transcriptional regulator in addition to having a direct role as a methyl acceptor during DNA repair. Thus, the up-regulated expression of the *ada *gene positively affects cell adaptation of alkylation damage by MMS in *E. coli *W3110 strain. The high level of *ada *expression, together with high level of 3-methyladenine-DNA glycosylase (encoded by the *alk*A gene), might be sufficient to repair these lesions, making high-level expression of the *aid*B and *alk*B genes unnecessary at 0.5 h after MMS treatment. This coordinated expression of the *alk*A and *ada *genes is noteworthy in that the two gene products repair different types of alkylation damage by different mechanisms, as illustrated [21]. The linked regulation of these two proteins thus optimizes the repair of several diverse lesions that are likely to be formed in DNA by a single alkylating agent. However, it can be postulated that *ada *mutant strain express higher amounts of other genes involved in DNA repair systems, as well as two different 3-methyladenine-DNA glycosylases (*tag *and *alk*A) in order to compensate for its function. Recent studies have demonstrated the presence of a second DNA repair methyltransferase, encoded by the *ogt *gene, for the direct repair of alkylating lesions in *E. coli*, in which the *ada *gene has been inactivated by mutation [31]. This was consistent with our observation that the expression of the *ogt *gene was highly up-regulated at 0.5 h in the MMS-treated *ada *mutant cells, showing that the *ogt *gene is required for cell adaptation in the absence of the *ada *gene. In addition, the expression of the *alk*B gene continually increased in MMS-treated *ada *mutant strain, revealing that these genes can trigger the adaptive response to alkylating agents in the *ada *mutant strain.

Another reaction that operates by the direct reversal of damage in the DNA of the *ada *mutant strain at 0.5 h is that of the DNA photolyase, encoded by the *phr*B gene [32]. Other up-regulated genes and proteins involved in DNA repair [24] at 0.5 h in the *ada *mutant strain are endonuclease III and VIII (*nth*); exonulease III (*xth*A); endonuclease IV (*nfo*); mismatch repair (*vsr *and *mut*HL); cleaning of precursor pool (*mut*T); nucleotide excision repair (*uvr*ABCD, and *mfd*); and post-replication repair, SOS regulation and translesion synthesis (*rec*A, *lex*A and *umu*DC). Moreover, redox control of transcription (*sox*RS) and DNA ligase (*lig*) were moderately increased at 0.5 h in the *ada *mutant strain. Proteome analysis also indicated that RecA was significantly increased in the wild-type strain after MMS treatment and decreased afterwards. On the other hand, it was relatively rapidly and continually increased in the *ada *mutant strain after MMS treatment. These results indicate that the adaptive response is regulated partially by the SOS response, a complex, graded response to DNA damage that includes timely induction of gene products that block cell division and others that promote mutation, recombination and DNA repair. However, it has been reported that the adaptive response is distinct from previously characterized pathways of DNA repair, particularly from the SOS response [8,33]. These findings indicated that the adaptation did not lead to expression of SOS functions, since the *rec*A and *lex*A mutant cells that were unable to perform SOS repair could be adapted to alkylating agents, such as MNNG [8,33]. This is inconsistent with our result that showed high expression levels of genes involved in SOS response in the MMS-treated wild-type and *ada *mutant strains. Their expression levels in the *ada *mutant strain were the higher than the wild-type strain.

The up-regulated LexA regulon included DNA recombination and repair genes (*rec*AN and *ruv*AB), nucleotide excision repair genes (*uvr*ABD), the error-prone DNA polymerase genes (*umu*DC) and DNA polymerase II gene (*pol*B). Continued up-regulation of the LexA regulon suggests that blockage of DNA replication and/or DNA damage persists, leading to SOS signaling. These results indicate that SOS-induced levels of these gene products are needed for the adaptive response caused by MMS. In particular, other SOS-inducible gene products are required for efficient adaptive response in the absence of the *ada *gene to compensate for its role. For example, it was evident that DNA damage caused by MMS led to a significant induction of the *dna*NQ gene expression [34], suggesting a requirement for increased amounts of at least some DNA polymerase III holoenzyme subunits for recovery from the DNA damage caused by MMS. Our results are in agreement with the other findings and additionally show that enhanced amounts of at least some subunits of the DNA polymerase III holoenzyme (*dna*NT) might be necessary to repair DNA damage caused by MMS. The up-regulated DNA biosynthesis-related genes included the genes for chromosome replication (*dna*C) and DNA primase (*dna*G). However, these genes did not increase in MMS-treated wild-type cells. This result suggests that increased amounts of at least some subunits of DNA polymerase III holoenzyme are required for repair and recovery of MMS induced DNA damage, in agreement with the small number of polymerase molecules per cell.

Taken together, the increase in expression of these genes seems to be connected to the SOS response, and provides evidence that the adaptive response is a timely response that is tightly regulated in a coordinated fashion, through both positive and negative control by the SOS and other DNA repair systems. Interestingly, the adaptation of the *ada *mutant strain appears to occur within a narrow window in response to the level of SOS induction.

## Conclusion

*E. coli *responds to alkylation stress by activating sets of co-regulated genes that help the cell to maintain homeostasis. Overall, the transcriptional and translational responses of the *ada *mutant strain by alkylation damage are similar to those of the wild-type strain, but some differences between the strains were observed within a narrow window following MMS treatment. The *ada *mutant strain showed that the adaptive response mediated a strong induction of many genes involved in DNA replication, recombination, modification and repair. This indicates that the adaptive response in the *ada *mutant strain by MMS treatment is a tightly regulated response that functions in a timely and coordinated fashion and is controlled both positively and negatively by the SOS and other DNA repair systems. Secondary effects of increased expression of drug or antibiotic resistance genes were observed with up-regulation of many transporter-related operons for acquiring MMS toxicity resistance. An additional interesting observation is that the *ada *mutation resulted in derepression of bacterial chemotaxis and flagellar synthesis, which suggests an additional role of Ada as a negative transcriptional regulator for the expression of the genes involved in chemotaxis and flagellar synthesis, although the Ada regulator might have only a limited influence on cellular physiology under normal growth condition.

## Methods

### Bacterial strains

*E. coli *W3110 (derived from K-12, λ^-^, F^-^, prototrophic) and its *ada *mutant (WA; W3110*ada*::Km^r^) strains were used in this study. The mutant strain was constructed by disrupting the *ada *gene in the chromosome of *E. coli *W3110 by a homologous recombination system using λ Red recombinase [35].

### Culture conditions and MMS treatment

Cells were cultivated at 37°C and 250 rpm in 100 mL of Luria-Bertani (LB) medium (10 g/L tryptone, 5 g/L yeast extract, and 5 g/L NaCl) in 250-mL Erlenmeyer flasks. Cells grown for 15 h were diluted 1:100 in fresh LB medium and further cultured to an optical density at 600 nm (OD_600_) of 0.4. Methyl methanesulfonate (MMS; Sigma-Aldrich, St. Louis, MO, USA) was added to 0.04% v/v [20], and cells were collected at predetermined sampling times (0.5, 1.5 and 3.9 h) for the analyses of transcriptome and proteome. For comparison, both strains were also grown without MMS addition as controls. Cell growth was monitored by measuring the OD_600 _using a spectrophotometer (Ultraspec3000; Pharmacia Biotech, Uppsala, Sweden). When required, ampicillin (50 μg/mL) and/or kanamycin (35 μg/mL) were supplemented.

### DNA microarray analysis

All procedures including RNA preparation, cDNA labeling, DNA hybridization and data analysis were carried out as described previously [36]. GenePlorer TwinChip *E. coli*-6 K oligo chips (GT3001; Digital Genomics, Seoul, Korea) were used according to the manufacturer's protocol. The microarray images were obtained using the Axon Scanner (Axon, Inc., Union City, CA, USA), and analyzed using the GenePix 3.0 (Axon) and Genesis 1.5.0. beta 1 http://genome.tugraz.at softwares. Briefly, the signal intensities higher than the mean background intensities by 3-fold greater than the overall standard deviation were chosen. Global normalization was carried out by dividing each of fluorescence intensities by their sums. The expression level of each gene was normalized to the variance of 1. Duplicate replicates were carried out. DNA microarray data are available in Additional file 2. All DNA microarray data were also deposited in Gene Expression Omnibus (GEO) database (GSE16565).

### Real-time PCR analysis

Real-time PCR analysis was performed as described previously [36] with the iCycler iQ real-time PCR detection system (Bio-Rad) using the iQ SYBR Green Supermix (Bio-Rad). The *rrsB *gene was used as a reference gene for normalization, and the data were analyzed using the 2^-ΔΔ*C*^_T _method [37]. The amplicons were obtained using the following primer sets.

*ada*-for (5'-GAAACGCCTGTAACGCTGG-3')

*ada*-rev (5'-GGCTTTAGGCGTCATTCCG-3')

*alk*A-for (5'-TGGCGAACGGCTGGATGATT-3')

*alk*A-rev (5'-TTCAACGGCATACCTAACGCTTT-3')

*alk*B-for (5'-GCCCATTGATCCGCAAAC-3')

*alk*B-rev (5'-CTGGAAATCTGGATAGCCCG-3')

*aid*B-for (5'-GAACGGCTGAATCCCTTGAACTG-3')

*aid*B-rev (5'-TGAAAACGCACATCG TCCAGAC-3')

### Two-dimensional gel electrophoresis

Two-dimensional gel electrophoresis (2-DE) experiments were performed using the IPGphor IEF system (GE Healthcare Life Sciences, Chalfont St. Giles, UK) and Protean II xi Cell (Bio-Rad, Hercules, CA, USA) as described previously [38]. Cell extracts were obtained as reported previously [39]. The protein samples (200 μg) were applied to the Immobiline DryStrips (18 cm, pH 3-10 NL; GE Healthcare) using in-gel rehydration in an IPGphor (GE Healthcare) using five phases of stepped voltages from 200 to 8000 V with total focusing of 60 kV·h. The strips were then placed on 12% w/v SDS-PAGE gels prepared by the standard protocol [40]. Protein spots were visualized using a silver staining kit (GE Healthcare) and the stained gels were scanned by a UMAX PowerLook 2100XL Scanner (UMAX Technologies, Inc., TX, USA). PDQuest 2-D Analysis Software (Bio-Rad) was used to automate the process of finding protein spots within the image and to quantify the density of the spots on a percentage of volume basis. Features resulting from non-protein sources (e.g. dust particles and scratches) were filtered out and protein spots were normalized and pairwise image comparisons were performed. At least triplicate gels of each sample were analyzed. All protein spots exhibiting at least 2-fold differences between the samples were evaluated for statistical significance using the Student's *t*-test and all spots with *p *values of < 0.05 were matched with the corresponding spots on the silver stained images for identification using LC-MS/MS.

### LC-MS/MS and data analysis

For protein identification by the MS/MS analysis, samples were prepared as described previously [41]. Tryptic peptides (10 μL aliquots) were analyzed by a nano-LC/MS system consisting of an Ultimate HPLC system (LC Packings, Amsterdam, Netherlands) and a quadrupole-time-of-flight (Q-TOF) MS (Micromass, Manchester, UK) equipped with a nano-ESI source as described previously [39]. The MASCOT search server (version 1.8; http://www.matrixscience.com/) was used for the identification of protein spots by querying sequence of the trypsin digested peptide fragment data. Reference databases used for the identification of target proteins were UniProt Knowledgebase (Swiss-Prot and TrEMBL; http://kr.expasy.org/) and NCBI http://www.ncbi.nlm.nih.gov/.

## Authors' contributions

JHB carried out the transcriptome analysis. MJH carried out the proteome analysis. JSY participated in the protein sequence analysis. JHB, MJH and SYL designed the study and drafted the manuscript. All authors read and approved the final manuscript.

## Supplementary Material

Additional file 1 **Table S1**. Proteins and genes exhibiting significant quantitative differences at 0.5 h proteome and transcriptome profiles. *E. coli *W3110 and *ada *mutant strains were cultivated under MMS-treated and -untreated conditions. Click here for file

Additional file 2 **Transcriptome analysis data.** The expression levels of the genes in *E. coli *W3110 and its *ada *mutant strains at 0.5, 1.5 and 3.9 h after MMS treatment based on the corresponding untreated control. The differentially expressed genes more than 2-fold were regarded as up- or down-regulated genes and further classified based on functional categories at each time point. Click here for file
