# Supplementary material for: Transcriptome and proteome analyses of adaptive responses to methyl methanesulfonate in Escherichia coli K-12 and ada mutant strains
Source: BMC Microbiol. 2009 Sep 3;9:186. doi: 10.1186/1471-2180-9-186 (PMC2753364; doi:10.1186/1471-2180-9-186)
Supplement: Additional file 1 — Table S1. Proteins and genes exhibiting significant quantitative differences at 0.5 h proteome and transcriptome profiles. E. coli W3110 and ada mutant strains were cultivated under MMS-treated and -untreated conditions. [file 1471-2180-9-186-S1.doc]

**Table S1 - Proteins and genes exhibiting significant quantitative differences at 0.5 h proteome and transcriptome profiles.** *E. coli* W3110 and *ada* mutant strains were cultivated under MMS-treated and -untreated conditions.

| **Spot No.** | **Gene name^1^** | **Protein description^1^** | **Swiss-Prot Accession No.^a)^** | **p*I*^2^** | **Mw (Da)^2^** | **Sequence coverage (%)^2^** | **Fold difference^3^** | | | | | |
| --- | --- | --- | --- | --- | --- | --- | --- | --- | --- | --- | --- | --- |
|  |  |  |  |  |  |  | **WA/W** | | **W_MMS/W** | | **WA_MMS/WA** | |
|  |  |  |  |  |  |  | **P** | **T** | **P** | **T** | **P** | **T** |
| 1 | *asp*A | Aspartate ammonia-lyase | P0AC38 | 5.19 | 52323 | 19 | 0.56 | 0.77 | 1.8 | 2.6 | 1.1 | 2.1 |
| 2 | *pfl*B | Formate acetyltransferase 1 | P09373 | 5.69 | 85303 | 19 | 1.0 | 1.1 | 1.6 | 1.1 | 1.6 | 2.1 |
| 3 | *pfl*B | Formate acetyltransferase 1 | P09373 | 5.69 | 85303 | 23 | 0.71 | 1.1 | 0.83 | 1.1 | 2.2 | 2.1 |
| 4 | *pyr*G^4^ | CTP synthase | P0A7E5 | 5.63 | 60336 | 16 | W | 1.2 | 1.5 | 1.2 |  | 1.7 |
| 5 | *sdh*A | Succinate dehydrogenase flavoprotein subunit | P0AC41 | 5.85 | 64381 | 21 | 0.77 | 0.78 | 1.8 | 1.0 | 1.8 | 0.88 |
| 6 | *opp*A | Periplasmic oligopeptide-binding protein [Precursor] | P23843 | 6.05 | 60861 | 21 | W | 0.82 | 0.91 | 1.0 | 2.3 | 2.1 |
| 7 | *gua*B | Inosine-5'-monophosphate dehydrogenase | P0ADG7 | 6.02 | 51990 | 35 | 1.1 | 0.81 | 1.3 | 0.9 | 1.8 | 1.2 |
| 8 | *deg*P | Protease do [Precursor] | P0C0V0 | 8.65 | 49323 | 36 | 1.5 |  | 1.5 |  | 0.59 |  |
| 9 | *man*X | PTS system mannose-specific EIIAB component | P69797 | 5.74 | 35026 | 33 | 0.4 | 0.84 | 1.8 | 1.7 | 1.1 | 1.2 |
| 10 | *rml*A1 | Glucose-1-phosphate thymidylyltransferase 1 | P37744 | 5.39 | 32673 | 40 | 0.26 |  | 1.5 |  | 0.77 |  |
| 11 | *deo*C | Deoxyribose-phosphate aldolase | P0A6L0 | 5.50 | 27716 | 55 | 0.67 | 0.90 | 1.5 | 1.5 | 1.0 | 1.7 |
| 12 | *grc*A (*yfi*D) | Autonomous glycyl radical cofactor | P68066 | 5.09 | 14275 | 77 | W | 1.2 | 1.5 | 1.4 |  | 2.3 |
| 13 | *grc*A (*yfi*D) | Autonomous glycyl radical cofactor | P68066 | 5.09 | 14275 | 37 | W | 1.2 | 2.1 | 1.4 |  | 2.3 |
| 14 | *pgm* | Phosphoglucomutase | P36938 | 5.43 | 58324 | 6 | 4.5 | 1.7 | 1.0 | 1.5 | 1.5 | 2.6 |
| 15 | *ahp*F | Alkyl hydroperoxide reductase subunit F | P35340 | 5.47 | 56142 | 17 | 1.3 | 0.99 | 2.9 | 1.6 | 2.2 | 1.6 |
| 16 | *mtn*N | MTA/SAH nucleosidase | P0AF12 | 5.09 | 24339 | 52 | 1.0 |  | 1.7 |  | 1.8 |  |
| 17 | *adk* | Adenylate kinase | P69441 | 5.55 | 23571 | 41 | 2.1 | 0.66 | 1.0 | 1.0 | 1.5 | 1.9 |
| 18 | *eda* | KHG/KDPG aldolase | P0A955 | 5.57 | 22270 | 37 | 1.3 | 0.82 | 2.6 | 1.0 | 0.91 | 0.77 |
| 19 | *rbs*B | D-ribose-binding periplasmic protein [Precursor] | P02925 | 6.85 | 30931 | 39 | 0.67 | 0.65 | 0.50 | 0.7 | 1.0 | 1.4 |
| 20 | *dsb*A | Thiol:disulfide interchange protein dsbA [Precursor] | P0AEG4 | 5.95 | 23090 | 29 | 1.6 | 1.1 | 2.2 | 1.2 | 1.1 | 1.2 |
| 21 | *osm*Y | Osmotically-inducible protein Y [Precursor] | P0AFH8 | 6.32 | 21061 | 28 | WA | 1.2 |  | 0.8 | 1.2 | 1.7 |
| 22 | *nus*A | Transcription elongation protein nusA | P0AFF6 | 4.53 | 54837 | 58 | 1.2 | 0.93 | 0.77 | 0.9 | 1.6 | 1.9 |
| 23 | *ace*F | Dihydrolipoyllysine-residue acetyltransferase component of pyruvate dehydrogenase complex | P06959 | 5.09 | 66055 | 34 | 0.91 | 0.72 | 0.91 | 1.2 | 1.4 | 0.44 |
| 24 | *tre*C | Trehalose-6-phosphate hydrolase | P28904 | 5.51 | 63797 | 35 | 1.2 | 1.1 | 1.6 | 1.5 | 2.4 | 2.6 |
| 25 | *yjj*K | Uncharacterized ABC transporter ATP-binding protein yjjK | P0A9W3 | 5.43 | 62404 | 40 | 1.0 | 0.95 | 1.5 | 1.1 | 1.3 | 2.8 |
| 26 | *gln*S | Glutaminyl-tRNA synthetase | P00962 | 5.89 | 63438 | 44 | 1.2 | 1.1 | 1.3 | 1.8 | 1.0 | 1.0 |
| 27 | *suc*A | 2-oxoglutarate dehydrogenase E1 component | P0AFG3 | 6.04 | 104996 | 30 | 1.9 | 1.2 | 1.8 | 2.0 | 1.1 | 1.7 |
| 28 | *glp*A | Anaerobic glycerol-3-phosphate dehydrogenase subunit A | P0A9C0 | 6.20 | 58921 | 22 | 1.3 | 1.5 | 1.1 | 1.6 | 2.5 | 2.1 |
| 29 | *rec*A | Protein recA | P0A7G6 | 5.09 | 37950 | 46 | 2.2 | 0.99 | 6.7 | 2.0 | 4.4 | 3.5 |
| 30 | *tsf*^d)^ | Elongation factor Ts | P0A6P1 | 5.22 | 30404 | 38 | 2.1 | 0.51 | 6.4 | 1.1 | 8.6 | 0.5 |
| 31 | *nfn*B | Oxygen-insensitive NAD(P)H nitroreductase | P38489 | 5.80 | 23890 | 51 | 1.3 | 0.97 | 2.1 | 1.3 | 2.4 | 1.5 |
| 32 | *sod*A | Superoxide dismutase [Mn] | P00448 | 6.45 | 23083 | 40 | 1.2 | 1.2 | 1.5 | 1.3 | 1.1 | 1.0 |
| 33 | *fab*G | 3-oxoacyl-[acyl-carrier-protein] reductase | P0AEK2 | 6.77 | 25544 | 60 | 0.77 | 0.68 | 1.5 | 1.2 | 2.2 | 1.1 |
| 34 | *rcs*B | Capsular synthesis regulator component B | P69407 | 6.85 | 23656 | 33 | 1.1 | 0.97 | 1.0 | 1.1 | 2.5 | 2.0 |
| 35 | *acc*B | Biotin carboxyl carrier protein of acetyl-CoA carboxylase | P0ABD8 | 4.66 | 16676 | 23 | 0.83 | 0.66 | 1.1 | 0.6 | 0.83 | 3.5 |
| 36 | *che*A | Chemotaxis protein cheA | P07363 | 4.79 | 71338 | 37 | WA | 1.7 |  | 0.8 | 8.0 | 2.1 |
| 37 | *inf*B | Translation initiation factor IF-2 | P0A705 | 5.80 | 97290 | 13 | WA | 1.3 | W_MMS | 1.4 | 1.5 | 0.47 |
| 38 | *mfd* | Transcription-repair-coupling factor | P30958 | 5.79 | 129901 | 8 | WA | 1.6 | W_MMS | 1.9 | 3.0 | 2.8 |
| 39 | *rpo*C | DNA-directed RNA polymerase subunit beta' | P0A8T7 | 6.67 | 155063 | 17 | 1.5 | 0.91 | 1.5 | 1.1 | 0.77 | 0.52 |
| 40 | *inf*B | Translation initiation factor IF-2 | P0A705 | 5.80 | 97290 | 18 |  | 1.3 | 2.5 | 1.4 | 0.67 | 0.47 |
| 41 | *thr*S | Threonyl-tRNA synthetase | P0A8M3 | 5.80 | 73967 | 27 | 0.67 | 0.97 | 3.5 | 1.0 | 2.5 | 1.6 |
| 42 | *pyr*G^d)^ | CTP synthase | P0A7E5 | 5.63 | 60336 | 27 | WA | 1.2 |  | 1.2 | 2.3 | 1.7 |
| 43 | *gln*A | Glutamine synthetase | P0A9C5 | 5.26 | 51871 | 43 | WA | 0.81 |  | 1.2 | 0.67 | 1.2 |
| 44 | *gat*Z | Putative tagatose 6-phosphate kinase gatZ | P37191 | 5.50 | 47079 | 44 | 1.4 | 0.65 | 1.1 | 0.9 | 0.56 | 1.4 |
| 45 | *glp*D | Aerobic glycerol-3-phosphate dehydrogenase | P13035 | 6.97 | 56715 | 41 | 2.2 | 1.2 | 2.7 | 0.7 | 0.67 | 3.0 |
| 46 | *rps*B | 30S ribosomal protein S2 | P0A7V0 | 6.61 | 26727 | 61 | 1.7 | 0.67 | 3.1 | 0.8 | 2.6 | 1.6 |
| 47 | *gln*H | Glutamine-binding periplasmic protein [Precursor] | P0AEQ3 | 8.44 | 27173 | 54 | 0.91 | 0.75 | 1.5 | 1.0 | 0.77 | 1.2 |
| 48 | *ace*B | Malate synthase A | P08997 | 5.39 | 60236 | 40 | 1.1 | 0.71 | 1.5 | 1.2 | 1.0 | 1.7 |
| 49 | *pck*A | Phosphoenolpyruvate carboxykinase [ATP] | P22259 | 5.46 | 59606 | 54 | 1.2 | 0.9 | 1.7 | 1.0 | 1.5 | 1.6 |
| 50 | *suc*B | Dihydrolipoyllysine-residue succinyltransferase component of 2-oxoglutarate dehydrogenase complex | P0AFG6 | 5.58 | 43984 | 34 | 1.0 | 0.93 | 1.0 | 1.0 | 1.2 | 1.8 |
| 51 | *pep*Q | Xaa-Pro dipeptidase | P21165 | 5.60 | 50144 | 39 | 1.1 | 1.0 | 1.1 |  | 0.91 | 1.0 |
| 52 | *wrb*A | Flavoprotein wrbA | P0A8G6 | 5.59 | 20832 | 53 | 0.83 | 1.3 | 0.83 | 1.0 | 0.77 | 2.3 |
| 53 | *yjg*F | UPF0076 protein yjgF | P0AF93 | 5.36 | 13603 | 44 | 1.5 | 0.77 | 3.3 | 1.4 | 0.67 | 0.87 |
| 54 | *yga*U | Uncharacterized protein ygaU | P0ADE6 | 5.71 | 16053 | 43 |  | 0.90 |  | 0.6 |  | 3.0 |
| 55 | *yga*U | Uncharacterized protein ygaU | P0ADE6 | 5.71 | 16053 | 65 |  | 0.90 |  | 0.6 |  | 3.0 |
| 56 | *ald*B | Aldehyde dehydrogenase B | P37685 | 5.44 | 56270 | 41 | 1.8 | 1.4 |  | 1.3 | 1.0 | 1.9 |
| 57 | *dpp*A | Periplasmic dipeptide transport protein [Precursor] | P23847 | 6.21 | 60255 | 45 | 1.2 | 0.98 | 1.5 | 1.8 | 2.1 | 1.8 |
| 58 | *ytf*Q | ABC transporter periplasmic-binding protein ytfQ [Precursor] | P39325 | 6.67 | 34323 | 15 | 0.77 | 1.1 |  | 0.9 |  | 1.2 |
| 59 | *gar*R | 2-hydroxy-3-oxopropionate reductase | P0ABQ2 | 5.58 | 30640 | 40 | 0.45 |  | 1.3 |  | 1.2 |  |
| 60 | *gpm*A | 2,3-bisphosphoglycerate-dependent phosphoglycerate mutase | P62707 | 5.85 | 28539 | 34 | 1.7 | 1.4 | 1.8 | 1.5 | 1.1 | 1.1 |
| 61 | *ela*B | Protein elaB | P0AEH5 | 5.35 | 11299 | 36 |  | 1.2 |  | 0.9 |  | 1.8 |
| 62 | *yhb*O | Protein yhbO | P45470 | 5.27 | 18847 | 33 |  | 0.84 |  | 0.9 |  | 3.2 |
| 63 | *ace*E | Pyruvate dehydrogenase E1 component | P0AFG8 | 5.46 | 99606 | 40 |  | 0.5 |  | 1.2 |  | 0.38 |
| 64 | *htp*G | Chaperone protein htpG | P0A6Z3 | 5.09 | 71378 | 50 | 0.83 | 2.1 | 2.0 | 3.1 | 4.5 | 3.0 |
| 65 | *pro*S | Prolyl-tRNA synthetase | P16659 | 5.12 | 63653 | 52 | 0.67 | 0.38 | 2.0 | 1.6 | 0.45 | 0.68 |
| 66 | *yea*G | Uncharacterized protein yeaG | P0ACY3 | 5.63 | 74434 | 47 | 1.9 | 1.0 | 2.3 | 1.1 | 2.1 | 2.6 |
| 67 | *tas* | Protein tas | P0A9T4 | 6.27 | 38476 | 9 | 1.0 |  | 1.4 |  | 0.83 |  |
| 68 | *yqj*D | Uncharacterized protein yqjD | P64581 | 9.05 | 11045 | 15 | 1.1 | 1.3 |  |  | 1.0 | 1.3 |
| 69 | *fli*C | Flagellin | P04949 | 4.50 | 51265 | 62 | WA | 8.6 | 0.83 | 0.9 | 0.45 | 0.71 |
| 70 | *pan*B | 3-methyl-2-oxobutanoate hydroxymethyltransferase | P31057 | 5.15 | 28219 | 35 | 1.8 | 0.91 |  | 1.3 | 1.0 | 1.0 |
| 71 | *fab*I | Enoyl-[acyl-carrier-protein] reductase [NADH] | P0AEK4 | 5.58 | 27846 | 43 | 1.7 | 0.90 | 1.8 | 1.0 | 1.3 | 1.4 |
| 72 | *ydf*G | NADP-dependent L-serine/L-allo-threonine dehydrogenase ydfG | P39831 | 5.65 | 27232 | 21 | 0.4 | 0.78 | 0.59 | 0.9 | 1.5 | 1.1 |
| 73 | *sod*B | Superoxide dismutase [Fe] | P0AGD3 | 5.58 | 21253 | 37 |  | 0.64 | 0.67 | 1.0 | 1.2 | 1.4 |
| 74 | *fab*A | 3-hydroxydecanoyl-[acyl-carrier-protein] dehydratase | P0A6Q3 | 6.13 | 18957 | 11 | 1.2 | 1.0 | 0.77 | 0.9 | 1.5 | 1.2 |
| 75 | *che*Y | Chemotaxis protein cheY | P0AE67 | 4.89 | 14088 | 37 | WA | 0.98 |  | 1.0 | 0.83 | 0.99 |
| 76 | *mal*P | Maltodextrin phosphorylase | P00490 | 6.94 | 90466 | 44 | 0.67 | 0.74 | 1.5 | 1.0 | 1.5 | 1.7 |
| 77 | *tna*A | Tryptophanase | P0A853 | 5.88 | 52740 | 60 | 0.19 | 0.76 | 0.36 | 1.2 | 0.91 | 2.3 |
| 78 | *ans*B | L-asparaginase 2 [Precursor] | P00805 | 5.95 | 36828 | 53 | W | 0.8 | 0.91 | 2.3 |  | 2.3 |
| 79 | *cdd* | Cytidine deaminase | P0ABF6 | 5.42 | 31520 | 46 | 0.23 | 0.97 | 1.0 | 3.7 | 1.2 | 1.3 |
| 80 | *udp* | Uridine phosphorylase | P12758 | 5.81 | 27142 | 68 | 0.30 | 0.97 | 1.0 | 1.7 | 1.2 | 2.0 |
| 81 | *tig* | Trigger factor | P0A850 | 4.83 | 48163 | 8 | 0.45 | 0.62 | 1.0 | 1.2 | 0.38 | 0.6 |

1 The gene name, description of identified proteins and accession number are from the ExPASy Proteomics Server (http://kr.expasy.org/). The search is performed on the current UniProt Knowledgebase release (Swiss-Prot and TrEMBL).

2 Calculated p*I*, MW and sequence coverage are from MASCOT search results of Matrix science (http://www.matrixscience.com/) based on the genome information.

3 Fold differences were calculated from at least triplicate proteome experiments (P) and duplicate transcriptome experiments (T). All proteins are statistically significant with *p* values of < 0.05. Spots that were only detected in *E. coli* W3110 or *ada* mutant under MMS-untreated and –treated conditions were arbitrarily assigned a fold difference of: W, WA, W_MMS and WA_MMS, respectively.

4 Proteins show a different isoform on 2-D gels according to a given condition.
